# Supplementary material for: COZOID: contact zone identifier for visual analysis of protein-protein interactions
Source: BMC Bioinformatics. 2018 Apr 6;19:125. doi: 10.1186/s12859-018-2113-6 (PMC5889581; doi:10.1186/s12859-018-2113-6)
Supplement: Supplementary file 2 — Software build. Executable binary file of the software tool. (ZIP 118,784 kb) [file 12859_2018_2113_MOESM2_ESM.zip › cozoid/java/docs/beansbinding-1.2.1-doc.zip!/org/jdesktop/el/impl/parser/package-tree.html]

org.jdesktop.el.impl.parser Class Hierarchy (Beans Binding)


---


|  |  |  |
| --- | --- | --- |
| **Overview** \| **Package** \| Class \| Use \| **Tree** \| **Deprecated** \| **Index** \| **Help** | |  |
| **PREV**   **NEXT** | **FRAMES**    **NO FRAMES**     **All Classes** |  |


---


## Hierarchy For Package org.jdesktop.el.impl.parser

**Package Hierarchies:**: All Packages

---

## Class Hierarchy

- java.lang.Object
  - org.jdesktop.el.impl.parser.**AstValue.Target**- org.jdesktop.el.impl.parser.**ELParser** (implements org.jdesktop.el.impl.parser.ELParserConstants, org.jdesktop.el.impl.parser.ELParserTreeConstants)- org.jdesktop.el.impl.parser.**ELParserTokenManager** (implements org.jdesktop.el.impl.parser.ELParserConstants)- org.jdesktop.el.impl.lang.**ELSupport**
          - org.jdesktop.el.impl.parser.**SimpleNode** (implements org.jdesktop.el.impl.parser.Node)
            - org.jdesktop.el.impl.parser.**ArithmeticNode**
              - org.jdesktop.el.impl.parser.**AstDiv**- org.jdesktop.el.impl.parser.**AstMinus**- org.jdesktop.el.impl.parser.**AstMod**- org.jdesktop.el.impl.parser.**AstMult**- org.jdesktop.el.impl.parser.**AstPlus**
              - org.jdesktop.el.impl.parser.**AstBracketSuffix**- org.jdesktop.el.impl.parser.**AstChoice**- org.jdesktop.el.impl.parser.**AstCompositeExpression**- org.jdesktop.el.impl.parser.**AstDeferredExpression**- org.jdesktop.el.impl.parser.**AstDotSuffix**- org.jdesktop.el.impl.parser.**AstDynamicExpression**- org.jdesktop.el.impl.parser.**AstEmpty**- org.jdesktop.el.impl.parser.**AstFloatingPoint**- org.jdesktop.el.impl.parser.**AstFunction**- org.jdesktop.el.impl.parser.**AstIdentifier**- org.jdesktop.el.impl.parser.**AstInteger**- org.jdesktop.el.impl.parser.**AstLiteralExpression**- org.jdesktop.el.impl.parser.**AstNegative**- org.jdesktop.el.impl.parser.**AstNot**- org.jdesktop.el.impl.parser.**AstNull**- org.jdesktop.el.impl.parser.**AstString**- org.jdesktop.el.impl.parser.**AstValue**- org.jdesktop.el.impl.parser.**BooleanNode**
                                                  - org.jdesktop.el.impl.parser.**AstAnd**- org.jdesktop.el.impl.parser.**AstEqual**- org.jdesktop.el.impl.parser.**AstFalse**- org.jdesktop.el.impl.parser.**AstGreaterThan**- org.jdesktop.el.impl.parser.**AstGreaterThanEqual**- org.jdesktop.el.impl.parser.**AstLessThan**- org.jdesktop.el.impl.parser.**AstLessThanEqual**- org.jdesktop.el.impl.parser.**AstNotEqual**- org.jdesktop.el.impl.parser.**AstOr**- org.jdesktop.el.impl.parser.**AstTrue**
          - org.jdesktop.el.impl.parser.**SimpleCharStream**- java.lang.Throwable (implements java.io.Serializable)
              - java.lang.Error
                - org.jdesktop.el.impl.parser.**TokenMgrError**
                - java.lang.Exception
                  - org.jdesktop.el.impl.parser.**ParseException**
              - org.jdesktop.el.impl.parser.**Token**

## Interface Hierarchy

- org.jdesktop.el.impl.parser.**ELParserConstants**- org.jdesktop.el.impl.parser.**ELParserTreeConstants**- org.jdesktop.el.impl.parser.**Node**- org.jdesktop.el.impl.parser.**NodeVisitor**

---


|  |  |  |
| --- | --- | --- |
| **Overview** \| **Package** \| Class \| Use \| **Tree** \| **Deprecated** \| **Index** \| **Help** | |  |
| **PREV**   **NEXT** | **FRAMES**    **NO FRAMES**     **All Classes** |  |


---
